# B chromosome in *Plantago lagopus* Linnaeus, 1753 shows preferential transmission and accumulation through unusual processes

**DOI:** 10.3897/CompCytogen.11i2.11779

**Published:** 2017-05-22

**Authors:** Manoj K. Dhar, Gurmeet Kour, Sanjana Kaul

**Affiliations:** 1 School of Biotechnology, University of Jammu, Jammu-180006, INDIA

**Keywords:** Accumulation, drive, FISH, Molecular markers, Unreduced gametes

## Abstract

*Plantago lagopus* is a diploid (2n = 2x =12) weed belonging to family Plantaginaceae. We reported a novel B chromosome in this species composed of 5S and 45S ribosomal DNA and other repetitive elements. In the present work, presence of B chromosome(s) was confirmed through FISH on root tip and pollen mother cells. Several experiments were done to determine the transmission of B chromosome through male and female sex tracks. Progenies derived from the reciprocal crosses between plants with (1B) and without (0B) B chromosomes were studied. The frequency of B chromosome bearing plants was significantly higher than expected, in the progeny of 1B female × 0B male. Thus, the B chromosome seems to have preferential transmission through the female sex track, which may be due to meiotic drive. One of the most intriguing aspects of the present study was the recovery of plants having more chromosomes than the standard complement of 12 chromosomes. Such plants were isolated from the progenies of B chromosome carrying plants. The origin of these plants can be explained on the basis of a two step process; formation of unreduced gametes in 1B plants and fusion of unreduced gametes with the normal gametes or other unreduced gametes. Several molecular techniques were used which unequivocally confirmed similar genetic constitution of 1B (parent) and plants with higher number of chromosomes.

## Introduction

B chromosomes are dispensable genetic elements that do not recombine with the chromosomes of the standard complement (A chromosomes). Generally, Bs differ morphologically from A chromosomes. B chromosomes have been observed to be heterochromatic in nature, composed mainly of repetitive or genetically inert DNA (Jones et al. 2008b, Kumke et al. 2016). In some cases heterochromatin content of B chromosomes has been reported to be similar to that of A chromosomes (Jones and Houben 2003). B chromosomes contain no genes with major function necessary for the growth and development of the plant. However, Carchilan et al. (2009) isolated 16 putative B chromosome-associated transcripts in rye by cDNA-AFLP, which constitute 0.7% of the total transcripts, thereby suggesting presence of some important genes on B chromosome. The mode of inheritance of B chromosomes is non-Mendelian, irregular and they follow their own evolutionary pathway. Bs persist in the populations which is exemplified by their widespread occurrence in plants; angiosperms, gymnosperms, some ferns, bryophytes and fungi besides, animals, including mammals (Jones and Rees 1982).

B chromosomes have generally been considered as nuclear parasites since their mode of inheritance is autonomous and drive ensures their survival in the population. Drive is the property that qualifies the B chromosomes as selfish elements. The various mechanisms of drive (reviewed by Chiavarino et al. 1998) include: i) the suppression of meiotic loss especially when only one B chromosome is present, ii) non- disjunction at the second pollen mitosis, and iii) higher competitive ability of B chromosome carrying pollen grain. The drive can occur at any stage of life cycle and has accordingly been classified as pre-meiotic, meiotic and post-meiotic (Camacho et al. 2000). In pre-meiotic drive, B chromosomes increase in number in the germline cells and when the latter enter meiosis to form gametes, the mean number of B chromosomes increases. This type of pre-meiotic accumulation has been observed in *Locusta migratoria* Linnaeus, 1758 (Viseras et al. 1990) and *Crepis capillaris* (Linnaeus) Wallr., 1840 (Parker et al. 1989, 1990). Meiotic drive depends on the functional symmetry of meiotic products. There are reports on existence of meiotic drive in some grasshopper species (Camacho 2005). Post-meiotic drive occurs immediately after meiosis during the development of the male and female gametophyte (Jones 1991). The molecular mechanisms involved in drive were not known for long, but recently Banaei-Moghaddam et al. (2012) showed meiotic drive to be due to non-disjunction of chromatids of B chromosome.

The B chromosomes show unstable meiotic behaviour, but have preferential segregation to the nuclei, which form gametes (Jones and Houben 2003). Also in rye, non-disjunction of Bs and unequal spindle formation at first pollen mitosis are responsible for the accumulation and transmission of B chromosomes at a higher rate to the next generation (Banaei-Moghaddam et al. 2012). Many B chromosomes have transmission rates clearly higher than 50%, which leads to their accumulation in the subsequent generations (Jones et al. 2008b). Accumulation of B chromosomes has been reported through female sex track in several plants and animals (Hewitt 1973). In some cases the B chromosomes accumulate during male meiosis (Nur 1962), while in few organisms accumulation is through both the sexes.

Variation in transmission rate of B chromosomes is a common feature since these tend to be lost in some progenies while they increase in number in others. The genetic control of the transmission rate of B chromosomes has been demonstrated in some plant species (Bougourd and Plowman 1996, Puertas et al. 2000). It was suggested that in maize a single major gene located on A chromosome controls B chromosome transmission by acting in the haploid egg cell at the time of fertilization (Chiavarino et al. 2001). In rye, non-disjunction of Bs and unequal spindle formation at first pollen mitosis are responsible for the accumulation and transmission of B chromosomes at a higher rate to the next generation (Banaei-Moghaddam et al. 2012).

*Plantago* Linnaeus, 1753 is a large genus, of annual/perennial herbs and sub-shrubs, with a worldwide distribution. It is the only genus within the family Plantaginaceae and is based on about 200 species (Rahn 1996). *Plantago lagopus* is a small (about 30 cm tall), annual herb. It grows as a weed in the Mediterranean region. The diploid chromosome number of the species is 2n = 2x = 12. *P. lagopus* is genetically unstable which is reflected in the presence of aneuploidy (Dhar and Koul 1995). Dhar et al. (2002) reported a novel B chromosome in *Plantago lagopus*, whose main body is composed of 5S rDNA and has few 45S rDNA sequences at the ends. The authors presented the experimental evidence of *de novo* origin of novel B chromosome in *P. lagopus* through specific DNA sequence amplification. Using molecular cytogenetic techniques like FISH and Fiber-FISH, Kour et al. (2014) further characterized this chromosome and reported it to be a mixture of rDNA sequences and transposable elements.

In order to explore the mechanism of accumulation of B chromosome in *Plantago lagopus*, extensive crossing experiments were conducted. Based on the data so obtained, transmission of B chromosome through male and female sex tracks was calculated. These studies are expected to throw light on the existence of drive in B chromosome of *P. lagopus* besides, understanding the mechanism of perpetuation in the populations.

## Materials and methods

The data on male and female transmission of B chromosome was collected over a period of 5 years (2005–2010). Seeds of *Plantago lagopus* were sown in earthen pots during October, every year. Generally, the seeds germinated within 4-5 days. After about 2 months, the seedlings were transplanted to the experimental beds in the Jammu University Botanic Garden.

Before transferring to the soil, young seedlings were uprooted from the pots and root tips excised. The root tips were used for cytological studies to determine the presence of B chromosome(s). The root tips were stained with Feulgen stain and squashed in 1% acetocarmine. For meiotic studies, young floral buds were used for cytology. Slides were prepared using anthers from freshly fixed buds squashed in 1% acetocarmine. Three cytotypes were used for the present investigation; plants with standard complement of 12 chromosomes (0B), plants with one B chromosome in addition to the standard complement (1B) and plants with two B chromosomes in addition to the standard complement (2B). For Fluorescence in situ hybridization (FISH) the protocol of Dhar et al. (2002) was followed. Probes for 45S (pTa71) (Gerlach and Bedbrook 1979) and 5S ribosomal DNA (pPov1) (Dhar et al. 2002) were used for FISH. The signals were visualized using a Zeiss Axioskop microscope equipped with phase contrast and epifluorescence.

## Transmission

The progenies raised after selfing and crossing were analyzed to determine the mode of transmission of B chromosomes. To work out the transmission of B chromosomes through male and female sex tracks, crosses were attempted between the 0B, 1B and 2B plants. The seeds obtained from different crosses were sown in the pots during October every year. The chromosome number of progeny plants was determined from the seedlings.

### Single stranded DNA conformation polymorphism (SSCP) analysis

For SSCP analysis, PCR was performed using the 5S rDNA specific primers. PCR product (5µl) was denatured by adding the loading dye consisting of 95% formamide, 10% glycerol, 0.25% bromophenol blue, 0.25% xylene cyanol) followed by its immediate quenching on ice. The samples were electrophoresed on 6% polyacrylamide gel in 0.5X TBE. For staining polyacrylamide gels, silver staining protocol of Bassam et al. (1991) was followed.

### Sequence specific amplified polymorphism (SSAP) analysis

SSAP analysis was performed as per the protocol of Pearce et al. (1999). The primers selected for the analysis were MKD-4, MKD-5, MKD-9, MDK-11, ASW-8, ASW-9 and ASW-10 (Kour et al. 2009). A total of twelve different combinations were made using these primers (Suppl. material 1: Table 1). The PCR amplified fragments were electrophoresed on 6% polyacrylamide denaturing gel in 0.5X TBE followed by silver staining. Only reproducible bands were considered for scoring.

**Table 1.** Data on crosses among 0B (female) × 1B (male) plants.

Parent plants (0B × 1B)	Progeny plants			Total plants		Ratio
	0B	1B	2B	0B	B	0B:B
36-9-07 × 36-1-07	06	09	06	06 (28.5)	15 (71.4)	1:1.2
36-9-07 × 36-2-07	09	06	03	09 (50.0)	09 (50.0)	1:1
40-6-07 × 40-11-07	18	09	09	18 (50.0)	18 (50.0)	1:1
40-6-07 × 40-13-07	27	21	09	27 (47.3)	30 (52.6)	1:1.1
1M-4-06 × 1M-1-06	15	06	0	15 (71.4)	06 (28.5)	2.5:1
TOTAL	75	51	27	75 (49.01)	78 (50.9)	1:1.04

Percentage in parenthesis.

### Simple Sequence Repeat (SSR) analysis

For SSR amplification four primer pairs were used as given by Squirrell and Wolff (2001) for *Plantago major*, and ten other primers were tried (Suppl. material 1: Table 2). PCR amplification of SSR loci was performed in 20µl reaction mixture containing 20ng DNA, 1X PCR buffer, 2.5mM MgCl_2_, 0.5µmol each primer, 200µM dNTPs, 1U *Taq* polymerase (Fermentas, USA). The thermal cycling conditions were as follows: initial denaturation at 94°C for 4min followed by 35 cycles of 94°C for 30sec, 55-65°C for 30sec, extension at 72°C for 1min and final extension for 10min at 72°C. The amplification products were resolved on 10% polyacrylamide denaturing gel in 0.5X TBE and subsequently visualized by silver staining. For each microsatellite locus, size of the alleles was estimated by comparison with standard size. After scoring the allelic bands, other bands were also scored.

**Table 2.** Data on crosses among 1B (female) × 0B (male) plants.

Parent plants (1B × 0B)	Progeny plants			Total plants		Ratio
	0B	1B	2B	0B	B	0B: B
36-1-07 × 36-9-07	06	24	09	06 (15.4)	33 (84.6)	1:5.5
36-2-07 × 36-9-07	03	09	06	03 (16.6)	15 (83.33)	1:5
40-11-07 × 40-6-07	03	04	03	03 (30.0)	07 (70.0)	1:2.3
40-13-07 × 40-607	21	24	12	21 (36.8)	36 (63.2)	1:1.7
1M-1-06 × 1M-4-06	06	21	06	06 (18.2)	27 (81.8)	1:4.5
35-2-06 × 35-5-07	06	18	06	06 (20.0)	24 (80.0)	1:4
TOTAL	45	100	42	45 (24.06)	142 (75.93)	1:3.1

Percentage in parenthesis.

## Results

**Figure 1.**
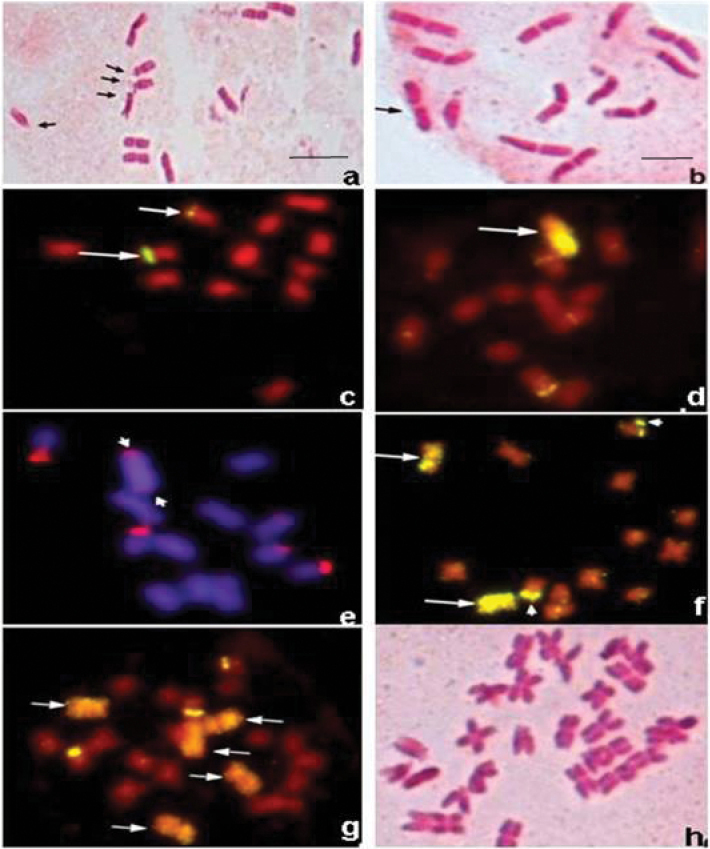
Root tip mitosis in different plants. **a** Metaphase spread showing 12 standard (A) chromosomes of *Plantago lagopus*. NOR bearing chromosomes have been marked **b** Metaphase spread showing 12 A and one B chromosome. Note the B chromosome is indistinguishable **c** Mapping of 5S rDNA sequences in 0B plant using FISH. Note the presence of 5S rDNA signals on two A chromosomes **d** Painting of B chromosome with 5S rDNA probe, besides signals of 5S rDNA on two A chromosomes **e** A metaphase spread containing one B chromosome probed with 45S rDNA, showing two additional NOR sites (arrow heads) **f** 5S rDNA probed metaphase spread showing 12 A and two B chromosomes **g** FISH of a metaphase spread of 23-chromosome plant revealing the presence of 5 B chromosomes **h** Metaphase spread showing 28 chromosomes. Scale bars: 10μm.

**Figure 2.**
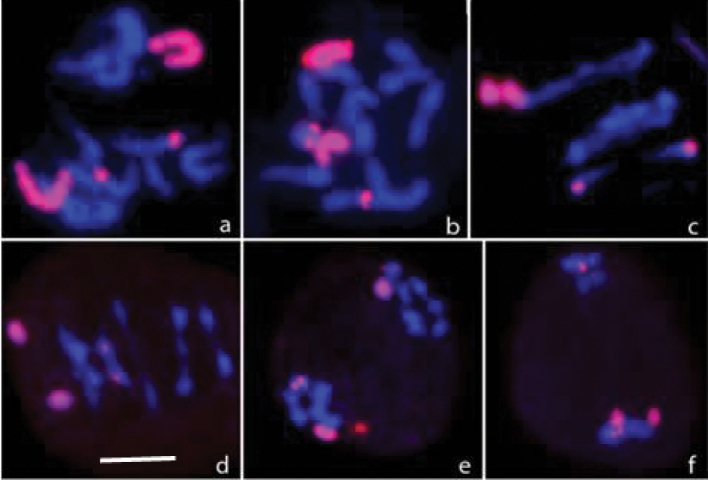
FISH on cells having 2 B chromosomes. Root tip metaphase cells at prometaphase (**a**) and metaphase (**b**) showing two B chromosomes. Segregation pattern of B chromosomes during meiosis (**c–f**). Note the two B chromosomes at the same pole (**f**). Scale bar: 10μm.

During the present investigation, about 531 plants were screened cytologically for the presence of the B chromosome (Fig. 1a, 1b), since it is not possible to distinguish plants with and without B chromosomes, morphologically. In *P. lagopus* the size of the B chromosome is almost equal to one of the A chromosomes, therefore, we used FISH with 5S rDNA probe, to identify the B chromosome(s) in 1B and 2B plants (Fig. 1c–f). As has been demonstrated in our earlier study (Dhar et al. 2002), the B chromosome gets completely painted when 5S rDNA is used as a probe (Fig. 1d). Therefore, in a 0B plant 5S rDNA signals were found only on two chromosomes (Fig. 1c). Similarly, 45S rDNA probe clearly identified the B chromosome, as the FISH signals were observed at the two ends of the B chromosome, besides the signals on the pair of NOR bearing chromosomes (Fig. 1e). In 2B plants, FISH with 5S rDNA probe clearly identified B chromosomes at somatic pro-metaphase and metaphase (Fig. 2a–b).

### Preferential transmission to progeny

The transmission of B chromosomes was ascertained through male and female sex tracks by attempting various reciprocal crosses. When 1B plant was used as a male, transmission rate was in accordance with the Mendelian expected ratio of 1:1 (p=0.05; χ^2^= 0.058) (Table 1). In the reciprocal crosses, when 1B plant was used as female parent, progeny based on a total of 187 plants was screened for chromosome number (Table 2); the ratio between the plants with and without B chromosome(s) was 3.1:1. Thus, it is clear that frequency of B chromosome bearing plants was higher than the Mendelian expected value (p < 0.05, χ^2^=50.30) when 1B chromosome plant was used as female parent. The deviation obtained from the Mendelian ratio was significant. These results clearly indicate that the B chromosome has preferential transmission through the female sex track.

### Meiotic behaviour of B chromosome

To understand the meiotic behaviour of B chromosome in 1B plants, a total of 1450 pollen mother cells from 145 plants were scanned at anaphase I and II. At metaphase-I the B chromosome remained as a univalent and at anaphase-I, in 1160 (80%) cells the B chromosome was present at one of the poles. In majority of the cases, the B chromosome seemed to have reached the pole earlier than the A chromosomes. At anaphase-II, the B chromosome divided into chromatids, which segregated to the poles. Similarly, in 2B plant at metaphase-I of meiosis, B chromosomes existed either as a bivalent (Fig. 2c) or two univalents (Fig. 2d). During anaphase-I the two chromosomes moved to the opposite or to the same pole (Fig. 2e, f). In 2B plants, 670 pollen mother cells were scanned, of which 490 cells showed B chromosomes at the poles earlier than the A chromosomes.

### Accumulation of B chromosomes

In some progenies of 1B plants, rarely, plants with very high number of chromosomes were observed, which included plants having 23, 26 and 28 chromosomes (Fig. 1g, h). FISH analysis of 23-chromosome plant with 5S rDNA probe revealed its chromosome constitution as 18A + 5B chromosomes. Similarly, plants with 26 and 28 chromosomes had 24A + 2B chromosomes and 24A + 4B chromosomes, respectively (figures not given). In other words, the B chromosomes were found in addition to triploid and tetraploid states.

### Origin of plants with 26 and 28 chromosomes

In order to trace the origin of plants with 2n = 26 and 2n = 28 chromosomes, three molecular markers were tried. The details are presented below:

### SSCP analysis

As shown in our earlier study (Dhar et al. 2002) and the present study, B chromosome is mainly composed of 5S rDNA sequences, therefore, SSCP analysis was carried out by targeting these sequences. For this purpose, DNA isolated from the selfed progeny plants of 1B and 2B parents (separately), including plants with 26 and 28 chromosomes, respectively, was used. The banding pattern of 1B (mother plant) and 26 chromosome progeny plant, 2B (mother plant) and 28 chromosome progeny plant showed 100% similarity. The remaining plants in the two progenies composed of 0B, 1B and 2B plants showed variable banding pattern (Suppl. material 3: Fig. S2).

### SSAP analysis

The SSAP exploits the insertional polymorphism of long terminal repeats (LTR retrotransposons) in the genome. In the present case SSAP analysis was used with twelve different primer combinations. It was observed that plant with 26 chromosomes showed 100% similarity with the 1B mother plant while other progeny plants showed polymorphism. Similarly, progeny plant with 28 chromosomes recovered from the selfed progeny of 2B plant showed 100% similarity with its mother plant as compared to other progeny plants.

### SSR analysis

For the present investigation we used 14 SSR primers. From SSR data generated demonstrated that 1B and 2B mother plants showed 100% similarity with the 26 (Suppl. material 4: Fig. S3a, b) and 28 (Suppl. material 4: Fig. S3c, d) progeny plants respectively, while differences were detected in other progeny plants.

The results obtained from the above three molecular markers clearly establish the maternal origin of 26 and 28 chromosome plants.

## Discussion

### Structure and behavior of B chromosome

In *Plantago lagopus* Dhar et al. (2002) demonstrated that the B chromosome is mainly composed of 5S rDNA sequences. Using FISH and reverse GISH techniques, the entire chromosome was found to get painted with 5S rDNA probe, while 45S rDNA sequences were localized at the two ends, just below the telomeric sequences. There are several reports on identification of B chromosomes using FISH (reviewed in Jones et al. 2008a). Most of the B chromosomes reported in different organisms are heterochromatic mainly due to the presence of repetitive DNA sequences made of satellite DNA, ribosomal DNA and transposable elements (Martis et al. 2012). Since 0B, 1B and 2B plants are indistinguishable morphologically (Dhar et al. 2002), therefore, in order to confirm the chromosomal status of the parents and the progeny plants, FISH with 5S rDNA probe was used in the present investigation.

The B chromosome does not pair or recombine with any A chromosome. B chromosomes, in general, have been reported to follow non-Mendelian mode of inheritance (Jones and Rees 1982) attributed to their irregular mitotic and meiotic behavior (Jones 1991). The meiotic behavior of the B chromosome in the pollen mother cells was very interesting. During anaphase, the B chromosome generally reached the poles earlier than A chromosomes. Thus, the B chromosome showed meiotic drive, which ensures its segregation and subsequent inclusion in the microspore mother cells.

### Transmission of the B chromosome

In the present case, transmission of the B chromosome was ascertained through male and female sex tracks by following the progenies of the reciprocal crosses. Interestingly, when 1B plant was used as a male, transmission rate was in accordance with the expected Mendelian ratio. The differences in segregation ratio observed among various cross combinations can be attributed to the heterozygous nature of *P. lagopus* - being a cross-pollinated species. On the other hand, when 1B plant was used as a female, there was significant deviation from 1:1 ratio; frequency of B chromosome bearing plants was higher than the Mendelian expected value. These results clearly indicate preferential transmission of B chromosome through the female sex track. According to Houben and Carchilan (2012), variation in transmission rate is a common feature of B inheritance, such that the Bs tend to distort Mendelian expectation in their favor. Their rate of transmission can be irregular with different levels of meiotic and post-meiotic drive or drag (Beukeboom 1994, Rusche et al. 1997). The drive through the female sex track can be explained on the basis of the fact that during female meiosis in plants, only one out of the four meiotic products survives. Therefore, in order to ensure inclusion in the surviving megaspore/egg, the B chromosome must be hosting a meiotic drive locus, as has been shown in *Mimulus* Linnaeus, 1753 - a phenomenon called female meiotic drive (Fishman and Saunders 2008). These authors have demonstrated that selfish chromosomal drive can be an important fitness determinant in natural populations. It can therefore be concluded that the B chromosome of *P. lagopus* is perpetuated preferentially due to drive, expressed during both male and female meiosis. The same is true of rye, where the drive happens in both male and female sex tracks (Jones and Houben 2003).

### Accumulation of B chromosomes

One of the important characteristics of B chromosomes is their accumulation in selfed or outcrossed progenies. In some species the accumulation is mainly due to non-disjunction of B chromosomes during pollen mitosis. The present case is perhaps the first in plants where the entire complement of the species gets duplicated in presence of a B chromosome. Earlier, Bidau (1987) reported the presence of macrospermatids (>diploid chromosome number) in B containing individuals of a grasshopper, *Dichroplus pratensis* Bruner, 1900. The formation of macrospermatids was attributed to nuclear fusion. In the present case, the plants with 23, 26 and 28 chromosomes were obtained from the selfed progenies of B chromosome bearing plants. The origin of these plants can be attributed to formation of unreduced gametes, followed by their fusion with other gametes or their endoreduplication and parthenogenetic development of the plant. Earlier, it has been postulated by Sharma et al. (1985) that in *P. lagopus* unreduced egg of the trisomic mother plant may have developed parthenogenetically giving rise to plants with 13 chromosomes. The authors reported formation of aneutriploid individuals with 19 chromosomes (Sharma et al. 1985) in the progeny of a cross between a trisomic and disomic plants. Similarly, Bhan et al. (1990) reported an autotetraploid in *P. lagopus* isolated from an experimental population, which could be the result of fusion of unreduced gametes.

Formation of unreduced gametes has been reported in many plants and has been proposed as an important mechanism for origin of polyploids (Mason et al. 2011, Mason and Pires 2015).

### Molecular markers in tracing origin of 26 and 28 chromosome plants

To substantiate the proposed mode of origin of plants with 26 and 28 chromosomes, recovered from selfed progeny of B chromosome plants, recourse was taken to molecular markers namely SSCP, SSAP and SSR. For SSCP analysis we targeted those sequences, which are present on the B chromosome. The technique was used to check whether Single Nucleotide Polymorphism exists among 1B and 2B mother plants and their progeny plants, including 26 and 28 chromosome plants. The SSCP pattern of 1B (mother) and 26 (progeny) plant showed 100% similarity, as compared to rest of the progeny plants. Similarly 2B (mother) and 28 chromosome progeny plants showed monomorphic pattern of bands, while in other 14 chromosome plants polymorphic pattern was observed. Thus SSCP analysis of 5S rDNA showed that the mother plants and the higher chromosome plants are 100% similar, thereby suggesting that they must have originated from the maternal genome. The experiments were repeated 3-4 times, however, similar SSCP pattern was observed in the mother plant and the offsprings with 26 and 28 chromosomes. SSCP technique is known to detect variation due to SNPs. Recombination is known to affect the SNPs; the SNP variation is less in regions of low recombination while it is more in high recombination regions (Brumfield et al. 2003). Recombination frequencies vary due to several genetic and non-genetic factors such as sex, the genetic background, genes and structures involved in meiotic recombination, age, irradiation, chemicals, nutrient salts and antibiotics (Barth et al. 2000). Occurrence of such processes more frequently during sexual reproduction in comparison to asexual reproduction, will lead to generation of SNPs in the former. This gets exemplified in the present case, by detection of large number of SNPs among progeny plants bearing 1B and 2B chromosomes than in 26 or 28 chromosome plants.

SSAP has also been used for the recombination studies in the selfed progeny plants of *Pisum* Linnaeus, 1753 (Jing et al. 2007). The marker has been used to detect the variation at genic level due to recombination and the combination of male and female genome mutation. Similarly, the inheritance of the B chromosome at the genome level has been analyzed using the SSR markers in *Brassica* sp. Linnaeus, 1753 (Navabi et al. 2011). SSAP and SSR used in the present case have also shown the same pattern in case of mother and the progeny plant having higher chromosome number. The molecular data does not support occurrence of recombination events in the origin of 26 and 28 chromosome plants, therefore, it can be presumed that the latter have originated through a two step process; formation of unreduced gametes in the parent, followed by parthenogenetic development of unreduced gametes, as has been proposed by Sharma et al. (1985).

### Unreduced gametes and parthenogenetic development

Apomixis, or clonal propagation by seed, has been reported in many genera of higher plants following the gametophytic apomixis (Carman 1997). Plants arising from apomixis retain the maternal genotype. The main components of apomixis include unreduced gamete formation and parthenogenetic development (Koltunow and Grossniklaus 2003), which are also exemplified by the plants studied in present investigation. The genes and pathways involved in gametophytic apomixis have not been discovered as yet and are the subject of intense research (Hand and Koltunow 2014). Polyploidy is the common feature of almost all apomicts (Roche et al. 2001). B chromosomes have been documented in apomictic species (Jones and Rees 1982), however, there is no report on apomixis gene(s) necessarily residing on a B-chromosome (Roche et al. 2001). In an animal species, B-chromosomes were found in polyploid individuals reproducing by pseudogamous parthenogenesis but were conspicuously absent in the diploid sexual individuals (Beukeboom et al. 1998).

Preferential transmission of B chromosomes and occurrence of plants with high chromosome numbers (2n =23, 26, 28) can have serious implications in the evolution of *Plantago lagopus* genome and the speciation. Recently, in *Arabidopsis thaliana* (Linneaus) Heynh., 1842, unreduced gamete-producing mutants, on account of defects in the meiotic cell cycle machinery, have been identified which has further advanced our understanding of the mechanisms behind unreduced gamete formation (Brownfield and Kohler, 2011). In the present case, plants with 23, 26 and 28 chromosomes were isolated in the progenies of the plants carrying B chromosome(s), therefore, it can be presumed that some DNA element located on B chromosome is activating the gene(s) promoting unreduced gamete formation, which is (are) located on A chromosome. Similar observations have been made in rye (Carchilan et al. 2009). However, more intense molecular studies need to be conducted to identify the gene/genes responsible in *P. lagopus*.

## Funding

This work was supported by Department of Science and Technology, Government of India (No. SR/SO/PS-07/2001), DST-DAAD (No. INT/FRG/DAAD/P-186/2009) and CSIR (No. 9/100(0169/2K12-EMR-I).

## Supplementary materials

Supplementary material 1 Tables S1 and S2 Data type: Nucleotide sequences Explanation note: Table S1: Sequences of primers used for SSR analysis. Table S2: The twelve different primer combinations used in the study. This dataset is made available under the Open Database License (http://opendatacommons.org/licenses/odbl/1.0/). The Open Database License (ODbL) is a license agreement intended to allow users to freely share, modify, and use this Dataset while maintaining this same freedom for others, provided that the original source and author(s) are credited.Manoj K. Dhar, Gurmeet Kour, Sanjana Kaul

Supplementary material 2 Figure S1 Data type: Images Explanation note: SSCP profile of 5S rDNA amplified from various plants. M = 100bp ladder. Lane1: 1B mother plant. Lane 2: progeny plant with 2n = 26 chromosomes. Lanes 3, 4: progeny plants (1B). Lane 5: 2B mother plant. Lane 6: progeny plant with 2n = 28 chromosomes. Lanes 7-9: progeny plants (2B). The band pattern of higher chromosome plants completely matches that of the mother plants as indicated by arrows. This dataset is made available under the Open Database License (http://opendatacommons.org/licenses/odbl/1.0/). The Open Database License (ODbL) is a license agreement intended to allow users to freely share, modify, and use this Dataset while maintaining this same freedom for others, provided that the original source and author(s) are credited.Manoj K. Dhar, Gurmeet Kour, Sanjana Kaul

Supplementary material 3 Figure S2 Data type: Images Explanation note: **S2a**: SSAP profile of 1B mother plant and progeny plants. M = 100bp ladder. Lane1: 1B mother plant. Lane 2: progeny plant with 2n = 26 chromosomes. Lanes 3-8: 1B progeny plants. **S2b**: SSAP profile of 2B mother plant and progeny plants. M = 100bp ladder. Lane 1: 2B mother plant. Lane 2: progeny plant with 2n = 28 chromosomes. Lanes 3-8: 2B progeny plants. This dataset is made available under the Open Database License (http://opendatacommons.org/licenses/odbl/1.0/). The Open Database License (ODbL) is a license agreement intended to allow users to freely share, modify, and use this Dataset while maintaining this same freedom for others, provided that the original source and author(s) are credited.Manoj K. Dhar, Gurmeet Kour, Sanjana Kaul

Supplementary material 4 Figure S3 Data type: Images Explanation note: **S3a, b**: SSR profile of 1B mother and its selfed progeny plants. M = 100bp ladder. Lane1: 1B mother plant. Lane 2: progeny plant with 2n = 26 chromosomes. Lanes 3- 26: 1B chromosome containing progeny plants. **S3c, d**: SSR profile of 2B and its selfed progeny plants. M = 100bp ladder. Lane1: 2B mother plant. Lane 2: progeny plant with 2n = 28 chromosomes. Lanes 3- 14: 2B chromosome containing progeny plants. Note exactly similar band pattern of mother and higher chromosome progeny plants. This dataset is made available under the Open Database License (http://opendatacommons.org/licenses/odbl/1.0/). The Open Database License (ODbL) is a license agreement intended to allow users to freely share, modify, and use this Dataset while maintaining this same freedom for others, provided that the original source and author(s) are credited.Manoj K. Dhar, Gurmeet Kour, Sanjana Kaul
