# Supplementary figures and images for: B chromosome in Plantago lagopus Linnaeus, 1753 shows preferential transmission and accumulation through unusual processes
Source: Comp Cytogenet. 2017 May 22;11(2):375–92. doi: 10.3897/CompCytogen.11i2.11779 (PMC5596978; doi:10.3897/CompCytogen.11i2.11779)

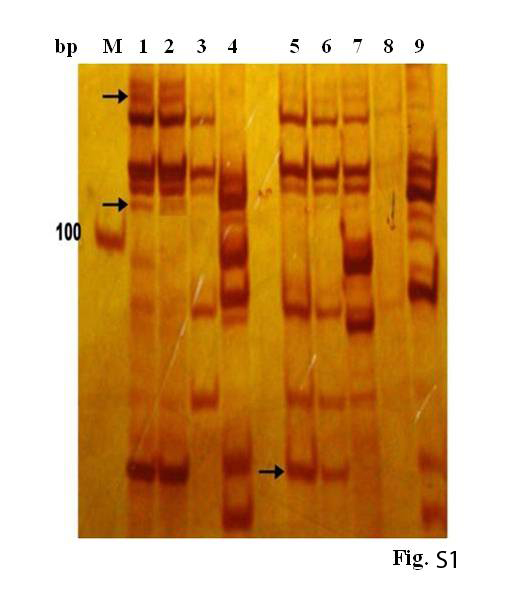

Supplement: Supplementary material 1 — Tables S1 and S2 [file comparative_cytogenetics-11-375-s001.jpg]

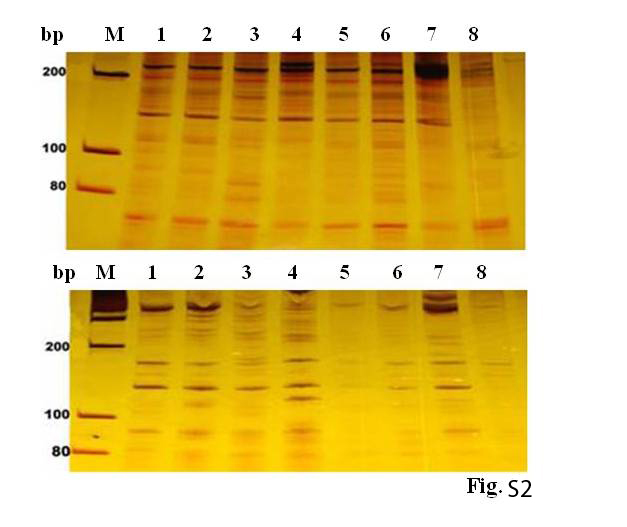

Supplement: Supplementary material 2 — Figure S1 [file comparative_cytogenetics-11-375-s002.jpg]

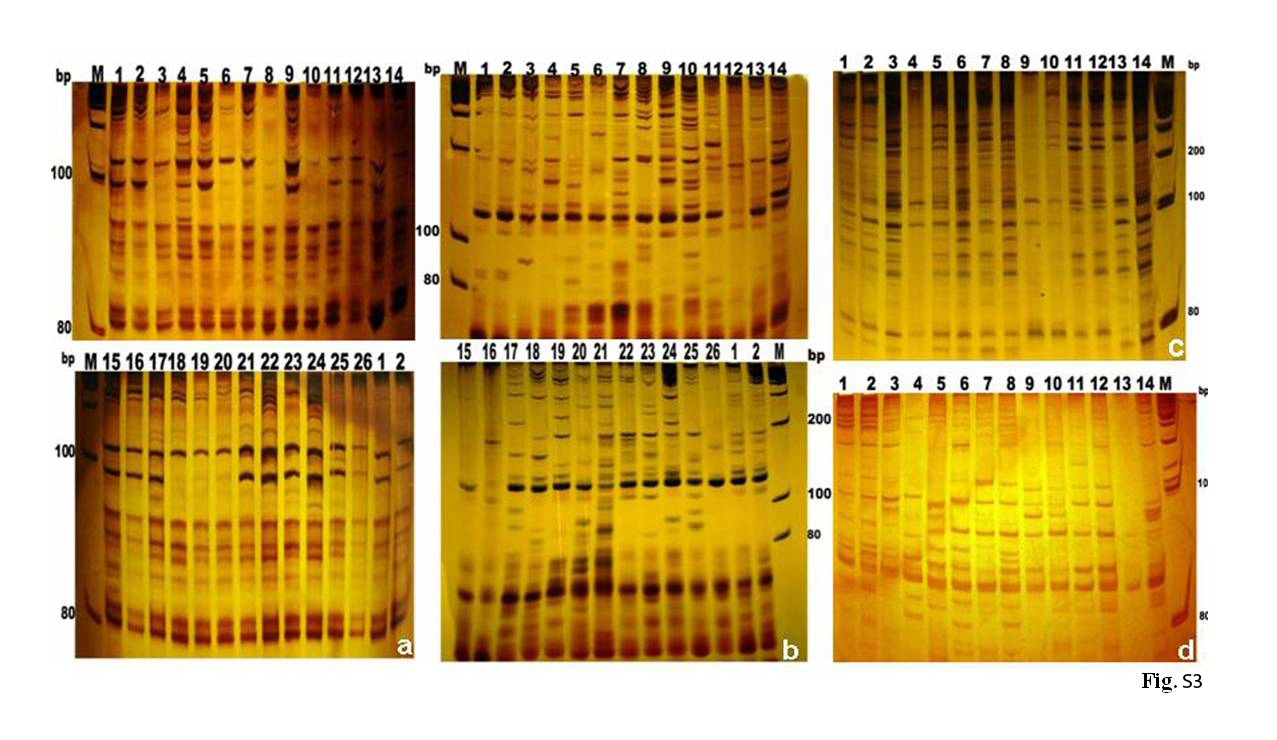

Supplement: Supplementary material 3 — Figure S2 [file comparative_cytogenetics-11-375-s003.jpg]
