# Supplementary material for: B chromosome in Plantago lagopus Linnaeus, 1753 shows preferential transmission and accumulation through unusual processes
Source: Comp Cytogenet. 2017 May 22;11(2):375–92. doi: 10.3897/CompCytogen.11i2.11779 (PMC5596978; doi:10.3897/CompCytogen.11i2.11779)
Supplement: Supplementary material 4 — Figure S3 [file comparative_cytogenetics-11-375-s004.doc]

**Supplementary Table 1: Sequences** of primers used for SSR analysis

| **S. No.** | **Primer code** | **Primer Sequence** |
| --- | --- | --- |
| 1. | Plm1F Plm1R | 5′-ATATGAATTAGCCAACAAA-3′ 5′-CCAGCTCCAAGTCAAAGTA-3′ |
| 2. | Plm2F Plm2R | 5′-ATGGCATGAGTGGACCAGAT-3′ 5′-AAAAGCTGGGCACCTACAAA-3′ |
| 3. | Plm3F Plm3R | 5′-CCGAAGTTTGCAGAGAAACA-3′ 5′-TGCATTTCCACTCTCTCTCCT-3′ |
| 4. | Plm4F Plm4R | 5′-ATCAGTTGCTGCCCTCTTTG-3′ 5′-GCTGGTGCATTTTTCGAGAT-3′ |
| 5. | Mdm1F Mdm1R | 5′-CGCATGCTGACATGTTGAAT-3′ 5′-CGGTGAGCCCTCTTATGTGA-3′ |
| 6. | Mdm2F Mdm2R | 5′-AAACTGAAGCCATGAGGGC-3′ 5′-TTCCAATTCACATGAGGCTG-3′ |
| 7. | Mdm3F Mdm3R | 5′-ACCACATTAGAGCAGTTGAGG-3′ 5′-CTGGTTTGTTTTCCTCCAGC-3′ |
| 8. | Mdm4F Mdm4R | 5′-CCCACCAATCAAAAATCACC-3′ 5′-TGAAGTATGGTGGTGCGTTC-3′ |
| 9. | Mdm5F Mdm5R | 5′-GAAAGACTTGCAGTGGGGAGC-3′ 5′-GGAGTGGGTTTGAGAAGGTT-3′ |
| 10. | Mdm6F Mdm6R | 5′-CAAGGAAATCATCAAAGATTCAAG-3′ 5′-CAAGTGGCTTCGGATAGTTG-3′ |
| 11. | Mdm7F Mdm7R | 5′-TGACGAAATCCACTACTAATGCA-3′ 5′-GATTGCGCGCTTTTTAACAT-3′ |
| 12. | Mdm8F Mdm8R | 5′-AGCGTCCAGAGCAACAGC-3′ 5′-AACAAAAGCAGATCCGTTGC-3′ |
| 13. | Mdm9F Mdm9R | 5′-AACCAGATTTGCTTGCCATC-3′ 5′-GCTGGTGGTAAACGTGGTG-3′ |
| 14. | Mdm10F Mdm10R | 5′-CCCTCTTCAGACCTGCATATG-3′ 5′-ACTGTTTCCAAGCGATCAGG-3′ |

**Supplementary Table 2: The twelve different primer combinations used in the study**

| **S. No.** | **Primer combinations** |
| --- | --- |
| 1 | MKD4 & ASW8 |
| 2 | MKD4 & ASW9 |
| 3 | MKD4 & ASW10 |
| 4 | MKD5 & ASW8 |
| 5 | MKD5 & ASW9 |
| 6 | MKD5 & ASW10 |
| 7 | MKD9 & ASW8 |
| 8 | MKD9 & ASW9 |
| 9 | MKD9 & ASW10 |
| 10 | MKD11 & ASW8 |
| 11 | MKD11 & ASW9 |
| 12 | MKD11 & ASW10 |
